# Relaunching a Traditional Durum Wheat Product: New Cultivars and Introgression Lines Identified for Frike Making in Turkey

**DOI:** 10.3390/foods12163037

**Published:** 2023-08-12

**Authors:** Fethiye Özberk, Fernando Martínez-Moreno, Ljiljana Kuzmanović, Carla Ceoloni, İrfan Özberk

**Affiliations:** 1 Akcakale Vocational College, University of Harran, 63300 Sanlıurfa, Turkey; 2 Department of Agronomy, University of Seville, Citra de Utrera km1, 41013 Seville, Spain; fernan@us.es; 3 Department of Agriculture and Forest Sciences (DAFNE), University of Tuscia, 01100 Viterbo, Italy; kuzmanovic@unitus.it; 4 Department of Field Crops, University of Harran, 63300 Sanliurfa, Turkey; ozberki@harran.edu.tr

**Keywords:** early harvested grain, firik, functional food, frike yield and quality, *Thinopyrum*, traditional dish, wheat-alien introgression

## Abstract

Frike is an ancient and traditional food product prepared from early harvested whole wheat grain, particularly durum wheat (DW). Due to its many health beneficial effects, frike is considered a functional food. It is also a lucrative commodity, produced in various West Asian and North African countries and typically in Southeastern Turkey. However, no systematic assessment of the most-suitable genotypes for frike production in the region is available. This study aimed to carry out such an evaluation, based on frike yield, quality traits, marketing price, and profitability, on a set of 20 DW cultivars and DW-*Thinopyrum ponticum* introgression lines (ILs). The results based on a field trial performed in Gölbaşı (Adıyaman, Turkey) in the 2021–2022 season revealed the Turkish varieties Tüten-2002, Edessa, Artuklu, and Perre, together with the R5 IL to have the highest frike yields measured on 3 kg of roasted fresh spikes. The highest marketing prices were obtained by Turkish varieties Sariçanak-98, Burgos, Sümerli, and Artuklu, along with the R112 IL, excelling in quality traits. Considering all parameters, the Turkish cultivars Artuklu, Firat-93, and Sariçanak-98, besides the R112 IL, resulted in being the most-convenient genotypes for frike making, thus representing good candidates for maintaining cultural and genetic diversity in food production from a staple crop such as DW.

## 1. Introduction

Frike (also firik, frik, freekah, freekeh, farik, or fireek) is an ancient and traditional food product prepared from early harvested whole wheat grain. According to food lore, frike consumption dates back to 2300 Before Common Era (BCE) [1]. Frike became common in the east Mediterranean Basin and has long been a significant part of the food culture in some West Asian and North African (WANA) countries, such as Algeria, Egypt, Lebanon, Jordan, Morocco, Syria, Tunisia, and Turkey [2,3,4]. The great Ottoman traveller Evliya Çelebi indicated in his travelogue (1630–1676) that frike was consumed as “firik soup’’ [5] in some alms houses, dervish lodges, caravanserais, and monasteries.

For frike making, wheat is typically harvested when the grains are still tender and green, at a stage ranging from 75 to 89 of the Zadoks scale [6]. Upon harvest, the kernels are parched, roasted, dried, and rubbed. Frike is the name of the food process rather than of a specific grain type. However, it is made from wheat and most commonly durum wheat. Frike has a low glycaemic index, low cholesterol content, as well as a high content of iron, fibre, and lutein [3]. It is also a good source of fructo-oligosaccharides and other functional compounds [7]. For its many positive effects on consumers’ health, frike is considered a functional food. The accumulation of functional compounds in the kernel starts from the spike formation to the end of the milk stage of grain filling. Harvest at the dough stage of kernel development results in the capture of a high amount of fructan groups, which help increase the calcium and iron use efficiencies [8]. The antioxidant, physicochemical, and nutritional values of frike obtained from immature grains vary based on variety and maturity period [9]. Apart from antioxidant activity, the content of total phenolic compounds decreases during the maturation period [10].

Changes in grain composition during the maturation period greatly affect frike’s characteristics [11]. Due to the high sugar content of the grains, spikes harvested at the late milk stage of grain filling are preferred to fully mature grains [2]. Harvest stage is crucial for optimum frike production. Early harvest might result in a large number of carbonic spots on the roasted grains, derived from the effect of the flames; conversely, at late harvest, yellow grains might be obtained instead of green ones. The growing stage from 75–77 of the Zadoks scale seems to be the best for spike harvest for frike making.

Despite its huge marketing potential, farmers and consumers of frike are still not taking full advantage of it, despite its nutritional attributes. Annual world frike production ranges between 250,000 and 300,000 t [2,4,7]. This can be increased to a million tons via increasing international marketing opportunities to far-eastern countries such as Japan. Frike is a lucrative product, its marketing price being about three- to four-times higher than that of bulgur [7,12], another typical DW dish in WANA countries [12,13]. Frike is produced in some specific locations in Turkey, starting from Gölbaşı (Adıyaman), spreading in the southwest direction to Yavuzeli and Oguzeli (Gaziantep), and jumping to Reyhanlı in Hatay Province. There are no accurate figures for frike production in Turkey, estimated to be around 2000 t (Dagyudan, pers. comm.).

Durum wheat (*Triticum turgidum* L. ssp. *durum* Desf.; DW) is the currently preferred raw material for frike making, and the selection of genotypes with better frike quality is desirable. In the pre-historic era, people avoided wheat species with fragile spikes at maturity and grain shattering traits, such as wild diploid einkorn (*T. boeoticum*) or wild emmer (*T. turgidum* L. ssp. *dicoccoides*), for frike making [14]. There is no evidence that cultivated emmer (*T. turgidum* L. ssp. *dicoccum*) was used for frike making, probably due to its thin seed coat, affected by flame and resulting in large carbonic spots on the grains during roasting [15]. Thus, DW possesses the most-suitable attributes for frike making, with the best frike being made from large and hard grains [2]. Cultivars Sarıçanak-98 in Gölbaşı, besides Zenit and Diyarbakır-81 in Gaziantep, were the preferred ones by farmers for their frike characteristics, such as high frike yield, early grain-filling ability, drought and cold tolerance, and suitability for roasting. However, no systematic study of cultivars suitable for frike making in the region is available, nor has such a survey been extended to novel DW genotypes, carrying short and stable introgressions from wild wheat relatives (WWRs). This opportunity was offered by a shared set of DW lines among the partners of a PRIMA project (acronym: IMPRESA; see the Funding Section). The set consisted of 58 entries, including North African, Italian, and Turkish cultivated varieties, Turkish land races, and DW-WWR introgression lines (ILs), stably incorporating single or multiple chromosomal segments, mostly deriving from *Thinopyrum* species. The various *Thinopyrum* spp. introgressions confer to the recipient ILs effective resistance against several major DW diseases, as well as other valuable and novel attributes [16,17,18,19].

The objective of this study was to assess the suitability for frike making, along with the yield, quality, marketing price, and profitability, of a subset of 20 DW genotypes, previously selected from the 58 mentioned above on the basis of agronomic and morpho-physiological traits.

## 2. Materials and Methods

### 2.1. Plant Materials

Materials consisted of a total of 20 DW genotypes, including 12 Turkish cultivars (Entry Nos. 1, 3, 4, 5, 7, 8, 11, 13, 14, 16, 17, and 18 in all tables), 2 Turkish landraces (Entry Nos. 19 and 20), 1 Italian cultivar (Entry No. 2), 2 DW-*Thinopyrum ponticum* introgression lines, homozygous carriers of a 23%-long (R5 Hom+, Entry No. 6) or 28%-long (R112 Hom+, Entry No. 10) alien segment on their 7AL arms, respectively [16], as well as sib lines carrying no alien introgression (Entry Nos. 9, 12, and 15). The DW-*Thinopyrum ponticum* ILs, and so, their null sib lines, are near-isogenic, as they were isolated in self-progenies following repeated backcrosses to the recurrent cv. Simeto. The 20 lines had been tested in the previous growing season in Adıyaman in 2020-2021 and selected on the basis of their early-maturing ability, good early drought and cold tolerance, high grain-yielding ability, as well as specific yield components such as the number of grains per spike and grain weight per spike. High glume and palea thickness for roasting suitability (>0.2 mm) were considered as additional selection criteria of candidate lines. The two landraces, despite being relatively late-ripening, were characterised by thick and hairy glumes. Awned cultivars were also given priority for being easily flammable during roasting.

### 2.2. Sowing, Harvesting, and Frike Preparation

Field trials were set up in fallow land employing a randomised complete block design with 20 entries and three replications in the Gölbaşı location of Adiyaman Province in Southeast Anatolia (Turkey). They were planted on 10 November 2021 by a plot drill with a seed rate of 500 grains m^−2^. The plot size was 6 m × 6 rows (=7.2 m^2^) at planting and 5 m × 6 rows (=6 m^2^) at harvest. At sowing, 300 kg ha^−1^ of 20-20-0 N-P-K fertiliser was applied, and 150 kg ha^−1^ of urea was supplied at the early stem elongation stage. No irrigation was practiced. All other agronomical measures were taken, such as weed control and rodent management. The crop maturity stage was monitored daily by local farmers and the local extension office involved in the experimental activity. The appropriate harvest time for each entry was determined by squashing the grains.

**Figure 1:**
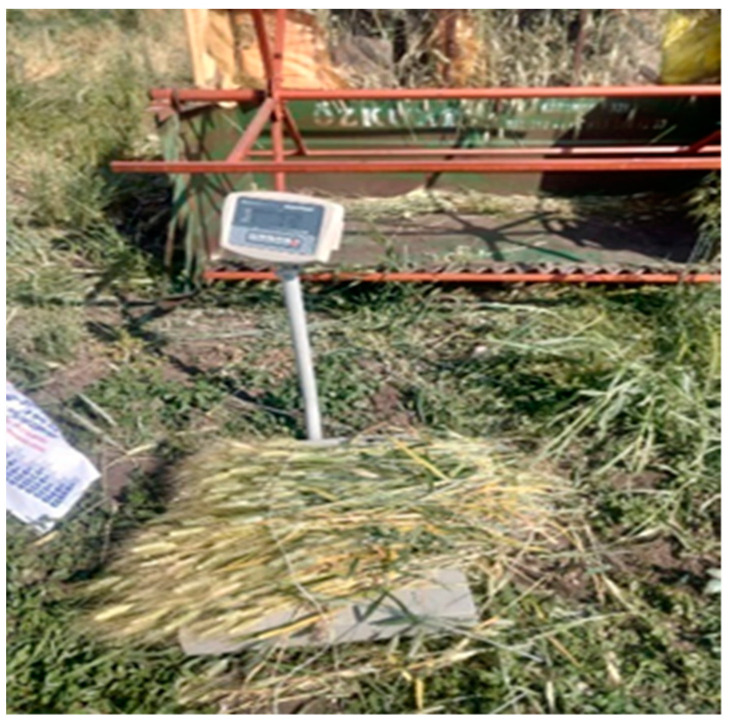
A sheaf of 3 kg of fresh spikes harvested from each plot prior to roasting.

**Figure 2:**
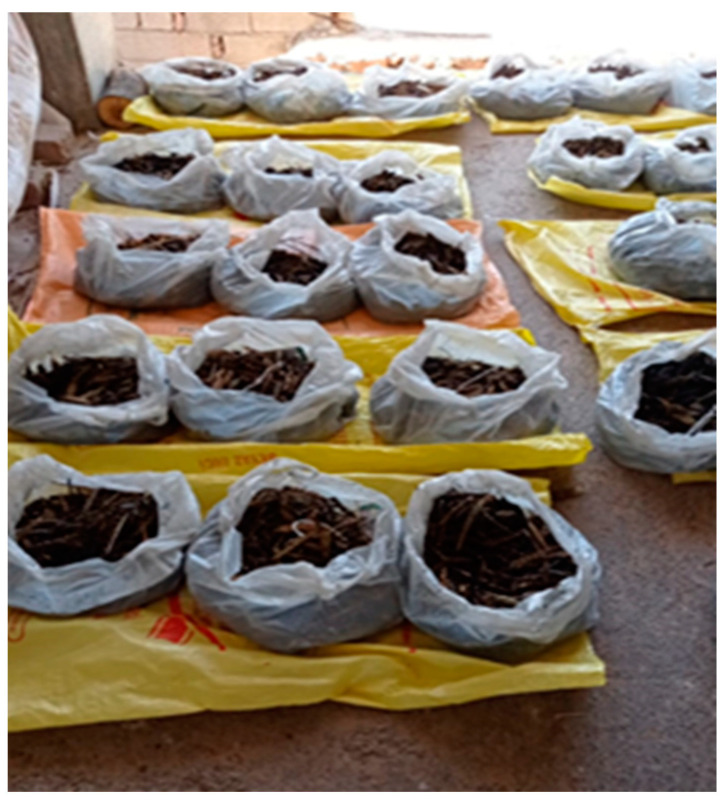
Roasted spikes of the various genotypes and replicates, let to further dry under a shade shelter for one week before threshing.

Upon the confirmation of the occurrence of the dough stage, 3 kg of spikes (including the peduncle) per plot and per replicate (a total of 9 kg of spikes from each entry) were trimmed (Figure 1) and immediately roasted, keeping the grain’s green colour stable in the experimental area. For roasting, performed by expert local farmers, open-air fires were set up in 2–3 small areas adjacent to the plots. Once the peduncle was removed, the spikes from each replicate were placed in a large iron sieve to be roasted over the fire. Roasted spikes were taken out of the sieve when their grains still showed a bright green colour. Since the grain moisture was generally still high, all frike samples were further dried under a shade shelter for one week (Figure 2).

**Figure 3:**
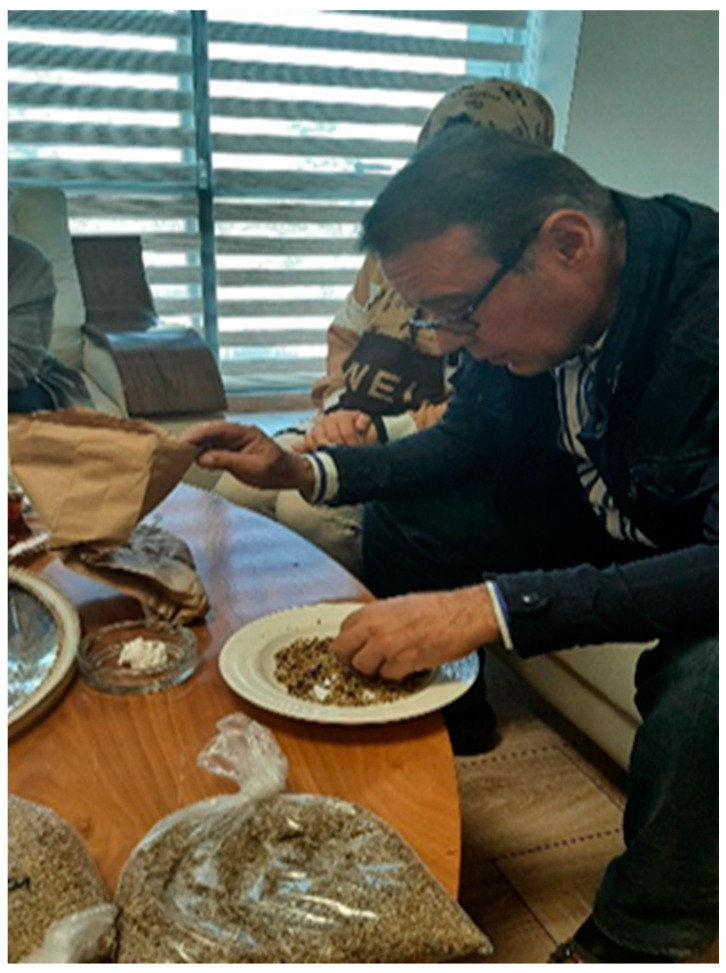
Frike inspection by a local purchaser for marketing price estimate.

Dried whole grain frike samples were threshed by a single plant thresher in the factory of the Olgunlar Seed Company in Adıyaman. The frike yield of the entries, i.e., the grain weight per 3 kg of harvested spikes, was scored. Frike samples of all replications were presented to a frike purchaser in a local commodity market (Figure 3).

### 2.3. Quality Analyses

Quality analyses were practiced at the seed quality laboratory of the local commodity market and at the Field Crop Department of the Faculty of Agriculture of Harran University (Turkey). Frike colour inspections of the samples were first made visually, employing a 1 to 5 scale (1: yellow, 2: cream, 3: light green, 4: green, and 5: dark green). Values of the L* (lightness, 100 = white; 0 = black), a* (-green; +red value), and b* (-blue; +yellow) colour coordinates were then scored by a Konica Minolta CR400 Chroma meter. A high L* value, combined with low a* and b* values, indicated a bright and green frike colour, as required for this product. Grain moisture (%), hectolitre weight (HLT, kg/hL), whole frike grain protein content (%), and wet gluten (%) were scored by a Perten 9500 analyser, calibrated according to the American Association of Cereal Chemistry (AACC) methods. The thousand-kernel weight (TKW) was scored as reported by Uluöz [20]. Raw cellulose (%) analysis was also performed, following the Haver–Boecker method [21].

Finally, frike samples of each cultivar were presented to a local purchaser for frike marketing price estimates (USD kg^−1^). Net returns were calculated by multiplying purchasing price × frike yield.

### 2.4. Statistical Analysis

Frike samples of all three replications per DW genotype were subjected to analysis of the yield and quality traits under study. Visual frike colour scores were transformed by the square root before ANOVA analysis. ANOVA was performed for the frike grain yield, frike colour (visual), L*, a*, and b* colour parameters, grain moisture, TKW, HLT, protein content, wet gluten, raw cellulose, and marketing prices, and a post hoc Tukey test (*p* < 0.05 level) was applied whenever a significant F-value was obtained.

Marketing price estimates of each entry were multiplied by the frike yields, and profitability (net return) per 3 kg of green spikes was calculated for each entry. Finally, the rank-sum method [22] was performed for the final selection of entries, considering all traits under study. The JMP 11 software was used for the statistical analyses.

## 3. Results

The 2021–2022 durum wheat-growing season had an average rainfall of 506 mm. The rainfall distribution throughout the season was not regular, with early drought resulting in late sowing (about one month) in autumn. Means and the groups of the characteristics under study are given in Table 1, Table 2 and Table 3. Raw data on which ANOVA was performed are reported in Table S1.

### 3.1. Quality Traits

#### 3.1.1. Frike Colour

Square-root-transformed data from visual inspections (Table 1) were subjected to ANOVA, and both replications (*p* ≤ 0.0001 ***) and entries (*p* ≤ 0.0006 ***) were highly significant. The coefficient of variation (CV) was 16.6%. Entries exhibiting the five best means for this frike colour criterion were R112 Hom+ (4.7), R5 Hom– (4.5), Sarıçanak-98 (4.0), Simeto (4.0), and R23 Hom– (3.3), respectively (Table S1). Genotypes sharing the background of the Italian cv. Simeto (see the Section 2) and, foremost, the DW-*Th. ponticum* R112 Hom+ introgression line gave dark-green frike.

As for the chroma meter indices, ANOVA indicated the absence of any significant source of variation for the L* parameter, despite a reliable CV of 8.35%. Devedişi provided the highest L* value (35.90), while Simeto gave the lowest (27.73). On the other hand, entries turned out to significantly differ for the a* value (*p* ≤ 0.0014 **), although a high CV (23.7%) was detected. Considering that the lowest values corresponded to the highest ranking for this trait, Devedişi, R112 Hom+, Simeto, Burgos, and Sariçanak-98 were the top five ranking entries, with 2.09, 3.54, 3.88, 4.02, and 4.10, respectively (Table 1). ANOVA indicated statistically significant differences among entries (*p* ≤ 0.039 *) also for the b* value (CV of 6.22%). With the lowest values representing, as for a*, the highest ranks, Simeto, R112 Hom+, Devedişi, Sarıbaşak, and R5 Hom+ occupied the first five positions in the ranking, giving 12.93, 13.72, 14.11, 14.49, and 14.53, respectively (Table 1).

#### 3.1.2. Additional Grain Quality Traits

Mean values for grain moisture (%) ranged from 8.8% to 9.36% (Table 2), with no significant difference. Statistically significant differences among entries (*p* ≤ 0.0001 ***) were observed for hectolitre weight (HLT). A low CV value (4.58%) indicated the high data reliability. The five entries with the highest HLT resulted in being R112 Hom+, Artuklu, Perre, R112 Hom–, and Sariçanak-98, with values of 78.16, 77.53, 77.26, 75.8, and 75.76 kg, respectively (Table 2). Similarly, ANOVA showed significant differences among entries for protein content (*p* ≤ 0.006 **), with a CV = 5.48% indicating the good reliability of the results. Firat-93, followed by cv. Simeto and three additional lines with a Simeto background (R112 Hom–, R112 Hom+, and R23 Hom–) were the top-five entries, giving protein percentages of 12.2, 12.1, 11.9, 11.63, and 11.43, respectively (Table 2). The tested entries significantly differed (*p* ≤ 0.0048 **; CV = 6.26%) also for the wet gluten that could be extracted from their grains. The top-five-ranking entries for this trait were the same as for protein content, namely Firat-93, Simeto, R112 Hom–, R112 Hom+, and R23 Hom–, which gave 25.4, 24.96, 24.6, 24.03, and 23.53% of wet gluten, respectively (Table 2). Rather unexpectedly, entries turned out to be non-significantly different in the ANOVA for thousand-kernel weight (CV = 15.39%). R5 Hom+ had the highest value (59.24 g), followed by its sib line (R112 Hom–), devoid of any alien introgression, with 55.06 g (Table 2). This indicated that, besides the good “background” value, an incremental contribution to TKW of R5+, especially compared with cv. Simeto (47.43), was due to the alien introgression. The Turkish landrace Devedişi gave the lowest figure (39.45 g). Finally, grains of the analysed materials showed no significant difference for raw cellulose amount (ANOVA *p* ≤ 0.08; CV = 17.67%). Scores ranged from 1.58 to 2.76. Akcakale-2000 gave the highest score with 2.76, and Artuklu, Haci Ali, Edessa, and R112 Hom– were in the second, third, fourth, and fifth ranking positions, giving figures of 2.50, 2.44, 2.25, and 2.14, respectively (Table 2).

### 3.2. Frike Yield

Although a 1:2 and 1:3 ratio between frike yield (g) and the 3 kg of fresh spike source (weight:weight, fully dry material) would be ideal (pers. comm.), the average ratio detected in the present analysis was nearly 1:6. Waste was in fact quite high, due to the contaminations of chaff and small stones remaining during roasting and the presence of carbonised kernels produced by over-roasting. ANOVA revealed that the entries were significantly different for this trait (*p* ≤ 0.006 **; CV = 15.66%). Tüten-2002, Edessa, R5 Hom+, Artuklu, and Perre ranked in the first five positions out of the 20 entries, with 679.91, 621.16, 620.03, 593.33, and 570.5 g/3 kg of green spikes, respectively (Table 3), corresponding to frike yield:fresh spikes ratios ranging from 4.4 (Tüten-2002) to 5.2 (Perre).

### 3.3. Marketing Price and Net Returns

Statistically significant differences (*p* ≤ 0.0001 ***) between the entries under study were observed for marketing price, although its CV was rather high (28.9%). After conversion of Turkish lira (TRY) kg^−1^ to USD kg^−1^, the five entries generating the highest marketing price resulted in being Sariçanak-98, R112 Hom+, Burgos, Sümerli, and Artuklu, giving USD 1.443, 1.428, 1.275, 1.049, and 1.026 kg^−1^, respectively (Table 3). The means of the marketing prices for each entry were multiplied by the frike yields obtained from 3 kg of fresh spikes. Net returns were calculated as USD 3 kg^−1^ of fresh spikes. Sarıçanak-98, Artuklu, Burgos, R5 Hom–, and Sümerli placed in the top-five positions, giving USD 0.653, 0.608, 0.581, 0.564, and 0.535 from 3 kg fresh spikes (Table 3).

### 3.4. Rank-Sum Analysis

The ranks of all the quality traits analysed were added, and the ranking of these values is given in Table 4. The ranks of all quality traits (A), frike yield (B), marketing price (C), and net return (D) were further evaluated, giving equal weight to the various criteria and applying a rank-sum analysis (Table 5). The results indicated that Artuklu, Firat-93, R5 (Hom–), Sariçanak-98, and R112 (Hom+) were the top-five genotypes and the most-convenient entries for frike making, giving the least sum of ranks (SR). Furthermore, Artuklu, Firat-93, and R5 (Hom–) were also particularly stable, giving low SR standard deviations (Table 5).

## 4. Discussion

To widen the spectrum of DW genotypes suitable for frike making in Turkey and potentially other countries with similar environmental conditions, several Turkish varieties and landraces, as well as an Italian DW cultivar and some DW-*Th. ponticum* introgression lines were tested. Various agronomic, quality, and market characteristics were evaluated, altogether providing a comprehensive appraisal of the most-suitable and satisfactory genotypes to meet the producers’ and consumers’ needs. The work was carried out following a “participatory” approach ([23] and the references therein), exploiting farmers’ experience and skill in various phases and considering their real needs, as well as the potential market. Harvest time was one of the relevant selection criteria in which local frike making farmers intervened. They preferred early-maturing lines, with longer staying-green period and the ability to escape from early drought. Green plant residues remaining in the field after fresh spike harvest are frequently used for silage production. Moreover, after silage making in late May, the empty field may be employed in the same season to grow cotton (Kozak, pers. comm.). Materials harvested in a timely manner generate shiny and pale-green frike. As soon as spikes are harvested, they must be roasted immediately; otherwise, they rapidly lose their original colour. Moreover, after roasting, they must be further dried in the shade or in a dark place until threshing. Among the genotypes exhibiting, on the basis of visual inspection, the desired green colour for frike making, the first ranking was the DW-*Th. ponticum* introgression line R112 Hom+ (4.66), followed by R5 Hom– (4.41), with a prevailing Simeto background and no alien introgression, and then the Turkish variety Sarıçanak-98 (3.92). It seems interesting to note that the IL R112 Hom+ is known to have high chlorophyll content and staying-green features, observed under different growing conditions [19,24,25,26]. The visual colour evaluation was confirmed by subsequent measurements of the L*, a*, and b* indices. Considering that a high L* value, combined with low a* and b* values, indicates a suitable bright and green frike colour, Sariçanak-98 was quite good regarding the L* parameter (33.34), confirming the results of Yıldırım and Atasoy [27]. As in their study, also in the present one, Sariçanak-98 and Burgos had a suitable a* value, whereas the b* value differed in the two analyses. For both the a* and b* values, the R112 Hom+ introgression line and the landrace Devedişi were the best-performing entries.

Regarding other grain attributes, hectolitre (HTL) and thousand-kernel weight (TKW) were quite high in the green-harvested grains, comparable to those of fully mature grains (see, e.g., [27]). Possibly, harvest was a little late for frike making; however, farmers preferred a little later harvest for some entries, accepting a little reduction of the green frike colour and marketing price, yet compensating their income with higher frike yield. This was the case of the R5 Hom+ introgression line, slightly later-maturing, but with a very good ranking for HTL and TKW (Table 2), as well as for frike yield (Table 3). The increased TKW values of the R5 Hom+ IL were in line with previous results showing this line to have enhanced TKW due to the presence of its alien segment (e.g., [19,26]).

Somewhat in contrast with the findings of Hamid and Omari [28], Yıldırım and Atasoy [27], and Akgün et al. [4], the protein content was not very high, being a little lower than those of fully mature grains. This suggested that the peculiar taste of frike could be likely attributed to other components, such as sugars, rather than to the high protein content [2,10,29,30,31].

Although non-significantly different among genotypes, the values for raw cellulose content, considered beneficial for health [7], were relatively high in the landrace Haci Ali and some well-established Turkish cultivars, such as Akcakale-2000, Artuklu, and Edessa. In the present study, the highest raw cellulose amount was 2.76%, lower than the average of 3.81% of a previous study [3], the difference being probably due to a somewhat later harvest of the present materials. In fact, Yang et al. [32] proved that immature grains contain a higher amount of raw cellulose than that of mature grains.

Shiny and green frike with the typical smoky flavour is given the highest marketing price. Large and harder grains receive a better marketing price offer [2]. The presence of carbonised grains, small soil pieces, or small stones with the same density of the frike and debris, as well as increasing yellow grain colour may reduce the marketing price sharply. The best purchasing price for shiny green frike was USD 1.87 kg^−1^, and the sale price on November 4, 2022, in the local market, was USD 2.41 kg^−1^. These, confirming previous results [7], were three-times the market price of bulgur. The net returns of genotypes indicated that neither frike yield, nor marketing price alone are good selection criteria for variety preference. Although Tüten-2002 ranked first in frike yield, due to its low marketing price, it ranked as 13th in net return (Table 3). The low market price assigned to this variety was likely caused by its low score for quality traits (ranking 20th out of 20 genotypes; Table 4), the composite “quality factor” having shown an overall good association with marketing price (Table 5). However, to minimise bias in variety evaluation, the rank-sum method was applied, which gave equal opportunity to frike quality traits, yield, marketing price, and net return to impact the comprehensive and reliable assessment of the materials under study.

Whereas, until recently, the Italian Cesare, Levante, and Zenit were the preferred DW varieties for frike making in the Sanliurfa (SE Anatolia) local commodity market [2], the present study provides stakeholders with a clear and comprehensive picture of several new and promising genotypes for relaunching frike production. Some such genotypes derive from national breeding activity, hence well adapted to local conditions, such as the Turkish varieties Artuklu, Firat-93, and Sariçanak-98, found to be the best-responding entries to all evaluation criteria. Furthermore, the biodiversity available for this relevant food product for the Turkish and potentially outside market can be apparently enriched with new candidates, consisting of DW-*Th. ponticum* introgression lines, particularly R112 Hom+, excelling in colour attributes, and R5 Hom+, with remarkable grain characteristics and frike yield. Their ability for transformation into this healthy and profitable product was here presented for the first time and adds value to the positive impact of their wild alien segment on the recipient DW performance, already known to be enhanced by the presence of resistance genes against leaf (*Lr19*) and stem rust (*Sr25*) of wheat [17], besides genes/QTL contributing to yield stability under heat and water-deficit stress [26]. Recently performed crosses between the above introgression lines and the best locally adapted varieties are expected to provide novel trait combinations for various exploitation purposes, including optimised frike production. Overall, this study represents a positive example in a wider perspective of maintaining cultural and genetic diversity in food production from a staple crop such as durum wheat [12,23].

## Figures and Tables

**Table 1:** Means of the tested durum wheat genotypes (entries) for frike colour parameters. Values in each column, ordered from highest to lowest, were analysed by the Tukey test at the *p* < 0.05 level. Values followed by the same letter(s) are not significantly different.

Entry No. (EN)	Entry Name	Visual Frike Colour		Colour Indices (Chroma Meter)					
		EN	1–5 Scale ^a^	EN	L*	EN	a*	EN	b*
1	Sarıbaşak	10	2.15 a	19	35.90	20	7.82a	4	16.82 a
2	Simeto	9	2.11 ab	17	34.58	16	6.90 ab	17	16.30 ab
3	Zühre	7	1.98 ab	15	33.59	14	6.33 ab	13	16.13 ab
4	Güneyyıldızı	2	1.98 ab	13	33.38	17	6.28 ab	11	16.02 ab
5	Fırat-93	15	1.82 ac	7	33.34	6	6.11 ac	16	15.96 ac
6	R5 (Hom+)	19	1.82 ac	4	33.17	12	6.09 ac	3	15.73 ac
7	Sariçanak-98	18	1.82 ac	3	32.81	3	6.04 ac	8	15.60 ac
8	Artuklu	11	1.73 ac	11	32.53	8	5.98 ac	7	15.36 ac
9	R5 (Hom–)	4	1.71 ac	20	32.19	5	5.73 ac	18	15.29 ac
10	R112 (Hom+)	5	1.66 ac	5	31.92	9	5.60 ac	14	15.29 ac
11	Sümerli	13	1.60 ac	9	31.88	4	5.50 ac	12	15.20 ac
12	R112 (Hom–)	14	1.60 ac	18	31.77	13	5.46 ac	15	14.98
13	A.Kale-2000	20	1.57 ac	8	31.68	1	5.05 ac	5	14.95 ac
14	Edessa	8	1.51 ac	12	31.33	15	5.02 ac	9	14.91 ac
15	R23 (Hom–)	6	1.41 ac	1	30.73	11	4.63 ac	20	14.82 ac
16	Tüten-2002	1	1.38 ac	10	30.58	7	4.10 ac	6	14.53 ac
17	Perre	3	1.38 ac	16	30.09	18	4.02 ac	1	14.49 ac
18	Burgos	12	1.13 bc	14	29.45	2	3.88 bc	19	14.11 ac
19	Devedişi	17	1.13 bc	6	29.03	10	3.54 bc	10	13.72 bc
20	Hacı Ali	16	1.11 c	2	27.73	19	2.09 c	2	12.93 c

^a^ For visual estimates of frike colour, square-root-transformed values are reported here (see the text), while raw data are in Table S1.

**Table 2:** Means of the tested durum wheat genotypes (entries) for various grain quality traits. Values in each column, ordered from highest to lowest, were analysed by the Tukey test at the *p* < 0.05 level. Values followed by the same letter(s) are not significantly different. TKW = thousand-kernel weight; HLT = hectolitre weight.

Entry No. (EN)	Entry Name	Moisture		TKW		HLT		Protein		Wet Gluten		Raw Cellulose	
		EN	%	EN	(g)	EN	(kg/hL)	EN	%	EN	%	EN	%
1	Sarıbaşak	9	9.36	6	59.24	12	78.16 a	5	12.23 a	5	25.4 a	13	2.76 a
2	Simeto	6	9.33	12	55.06	8	77.53 a	2	12.10 a	2	24.96 ab	8	2.50 ab
3	Zühre	3	9.33	16	51.14	17	77.26 a	12	11.90 a	12	24.60 ab	20	2.44 ab
4	Güneyyıldızı	14	9.30	10	50.38	10	75.80 a	10	11.63 ab	10	24.03 ac	14	2.25 ab
5	Fırat-93	15	9.26	15	49.76	7	75.76 a	15	11.43 ab	15	23.53 ac	12	2.14 ab
6	R5 (Hom+)	10	9.23	9	49.09	6	75.49 a	20	11.30 ab	20	23.16 ac	16	2.12 ab
7	Sariçanak-98	17	9.23	3	47.88	4	75.06 a	7	11.20 ab	9	22.95 ac	5	2.12 ab
8	Artuklu	1	9.23	5	47.80	3	75.00 a	18	11.20 ab	18	22.93 ac	19	2.10 ab
9	R5 (Hom–)	13	9.23	2	47.43	5	74.86 a	9	11.19 ab	7	22.90 ac	15	2.08 ab
10	R112 (Hom+)	8	9.16	17	47.20	16	74.84 a	8	11.16 ab	8	22.83 ac	3	2.03 ab
11	Sümerli	16	9.16	8	46.85	13	74.40 a	1	11.13 ab	6	22.78 ac	1	2.03 ab
12	R112 (Hom–)	11	9.13	20	45.30	11	74.26 a	6	11.12 ab	1	22.76 ac	18	2.01 ab
13	A.Kale-2000	2	9.10	1	44.86	1	74.06 a	14	11.06 ab	14	22.56 ac	9	1.98 ab
14	Edessa	4	9.10	13	44.83	2	73.60 a	13	11.03 ab	13	22.53 ac	2	1.95 ab
15	R23 (Hom–)	5	9.10	18	44.47	9	73.34 a	4	10.90 ab	4	22.16 ac	10	1.88 ab
16	Tüten-2002	18	9.10	14	43.67	14	72.63 a	11	10.86 ab	11	22.16 ac	17	1.87 ab
17	Perre	12	9.06	7	42.30	15	71.13 a	3	10.80 ab	3	22.00 ac	4	1.86 ab
18	Burgos	7	8.96	4	40.76	20	71.06 a	17	10.40 ab	17	20.93 bc	11	1.85 ab
19	Devedişi	19	8.93	11	40.20	18	70.43 a	16	10.14 ab	16	20.45 bc	7	1.59 b
20	Hacı Ali	20	8.80	19	39.45	19	56.90 b	19	9.8 b	19	19.66 c	6	1.58 b

**Table 3:** Means of entries for frike yield, marketing prices, and net return. Values in each column, ordered from highest to lowest, were analysed by the Tukey test at the *p* < 0.05 level. Values followed by the same letter(s) are not significantly different.

Entry No. (EN)	Entry Name	Frike Yield		Marketing Price		Net Return	
		EN	(g)	EN	(USD/kg)	EN	(USD/3 kg Fresh Spikes)
1	Sarıbaşak	16	679.91 a	7	1.443 a	7	0.653
2	Simeto	14	621.16 a	10	1.428 ab	8	0.608
3	Zühre	6	620.03 ab	18	1.275 ac	18	0.581
4	Güneyyıldızı	8	593.33 ab	11	1.049 ad	9	0.564
5	Fırat-93	17	570.50 ab	8	1.026 ad	11	0.535
6	R5 (Hom+)	5	560.50 ab	9	1.015 ad	5	0.531
7	Sariçanak-98	3	559.33 ab	2	0.984 ad	6	0.492
8	Artuklu	9	556.91 ab	5	0.948 ad	2	0.486
9	R5 (Hom–)	13	554.83 ab	1	0.808 ad	10	0.482
10	R112 (Hom+)	1	552.33 ab	19	0.801 ad	14	0.466
11	Sümerli	12	533.16 ab	6	0.794 ad	1	0.446
12	R112 (Hom–)	11	510.66 ab	14	0.751 ad	17	0.404
13	A.Kale-2000	4	502.33 ab	17	0.701 ad	16	0.382
14	Edessa	20	496.66 ab	12	0.626 bd	12	0.333
15	R23 (Hom–)	2	494.00 ab	20	0.576 bd	13	0.319
16	Tüten-2002	18	456.33 ab	13	0.576 bd	19	0.315
17	Perre	15	453.16 ab	16	0.564 bd	20	0.285
18	Burgos	7	451.66 ab	4	0.551 cd	4	0.276
19	Devedişi	19	394.66 ab	15	0.526 cd	3	0.266
20	Hacı Ali	10	338.83 ab	3	0.477 d	15	0.238

**Table 4:** Ranking of the analysed genotypes (entries) for frike major quality traits. TKW = thousand-kernel weight; HLT = hectolitre weight.

Entry No. (EN)	Entry Name	Frike Visual Colour	Colour Indices			HLT	TKW	Protein	Wet Gluten	Raw Cellulose	Sum of Ranks	Rank of Sum of Ranks
			L*	a*	b*							
1	Sarıbaşak	16	15	8	4	13	13	11	12	11	103	13
2	Simeto	4	20	3	1	14	9	2	2	14	69	4
3	Zühre	17	7	14	15	8	7	17	17	10	112	15
4	Güneyyıldızı	9	6	10	20	7	18	15	15	17	117	16
5	Fırat-93	10	10	12	8	9	8	1	1	7	66	3
6	R5 (Hom+)	15	19	16	5	6	1	12	11	20	105	14
7	Sariçanak-98	3	5	5	13	5	17	7	9	19	83	8
8	Artuklu)	14	13	13	14	2	11	10	10	2	89	9
9	R5 (Hom–)	2	11	11	7	15	6	9	7	13	81	7
10	R112 (Hom+)	1	16	2	2	4	4	4	4	15	52	1
11	Sümerli	8	8	6	17	12	19	16	16	18	120	17
12	R112 (Hom–)	18	14	15	10	1	2	3	3	5	71	5
13	A.Kale-2000	11	4	9	18	11	14	14	14	1	96	11
14	Edessa	12	18	18	11	16	16	13	13	4	121	18
15	R23 (Hom–)	5	3	7	9	17	5	5	5	9	65	2
16	Tüten-2002	20	17	19	16	10	3	19	19	6	129	20
17	Perre	19	2	17	19	3	10	18	18	16	122	19
18	Burgos	7	12	4	12	19	15	8	8	12	97	12
19	Devedişi	6	1	1	3	20	20	20	20	8	79	6
20	Hacı Ali	13	9	20	6	18	12	6	6	3	93	10

**Table 5:** Ranking of the analysed genotypes (entries) for frike quality traits, yield, and market features. STD = standard deviation.

Entry No. (EN)	Entry Name	Quality Traits (A)	Frike Yield (B)	Marketing Price (C)	Net Return (D)	Sum of Ranks (SR)	Rank of SR (EN)	STD of A, B, C, and D
1	Sarıbaşak	13	10	9	11	43	12	1.70
2	Simeto	4	15	7	8	34	7	4.65
3	Zühre	15	7	20	19	61	19	1.73
4	Güneyyıldızı	16	13	18	18	65	20	2.36
5	Fırat-93	3	6	8	6	22	2	2.06
6	R5 (Hom+)	14	3	11	7	35	8	6.23
7	Sariçanak-98	8	18	1	1	28	4	8.04
8	Artuklu	9	4	5	2	20	1	2.56
9	R5 (Hom–)	7	8	6	4	25	3	1.70
10	R112 (Hom+)	1	20	2	9	32	5	8.75
11	Sümerli	17	12	4	5	38	9	6.13
12	R112 (Hom–)	5	11	14	14	44	13	4.24
13	A. Kale-2000	11	9	16	15	51	16	3.30
14	Edessa	18	2	12	10	42	11	6.60
15	R23 (Hom–)	2	17	19	20	58	18	8.42
16	Tüten-2002	20	1	17	13	51	15	8.34
17	Perre	19	5	13	12	41	10	5.73
18	Burgos	12	16	3	3	34	6	6.55
19	Devedişi	6	19	10	16	51	14	5.85
20	Hacı Ali	10	14	15	17	56	17	2.98

## Data Availability

The data presented in this study are available within the article and Supplementary Material.

## Supplementary Materials

The following Supporting Information can be downloaded at: https://www.mdpi.com/article/10.3390/foods12163037/s1, Table S1: Raw data used for ANOVA analysis of tested traits, along with the mean and standard deviation (STD) of each trait in all entries.
