# Supplementary material for: Relaunching a Traditional Durum Wheat Product: New Cultivars and Introgression Lines Identified for Frike Making in Turkey
Source: Foods. 2023 Aug 12;12(16):3037. doi: 10.3390/foods12163037 (PMC10453173; doi:10.3390/foods12163037)
Supplement: Supplementary file 1 [file foods-12-03037-s001.zip › foods-2540852-supplementary.pdf]

Supplementary material

Relaunching a traditional durum wheat product: new cultivars and introgression lines identified for frike making in Turkey

Fethiye Özberk. Fernando Martínez-Moreno. Ljiljana Kuzmanović. Carla Ceoloni. İrfan Özberk

Table S1. Raw data used for ANOVA analysis of tested traits, along with mean and standard deviation (STD) of each trait in all entries.

Entry name	Entry No.	Replica	Frike yield (g/3 kg spikes)	Frike colour (1-5) Visual	L* (Chroma meter)	a* (Chroma meter)	b* (Chroma meter)	Grain moisture (%)	Hectolitre weight (kg/hL)	1000 kernel weight (g)	Protein (%)	Wet gluten (%)	Raw cellulose (%)
Sarıbaşak	1	1	506.5	2	29.87	4.71	14.11	9.4	74.2	42.4	11.4	23.4	2.42
		2	578.5	3	34.46	6.36	15.63	9.2	75.5	46.3	11.0	22.4	2.11
		3	572	1	27.87	4.08	13.75	9.1	72.5	45.9	11.0	22.5	1.56
		Mean	552.3	2.0	30.73	5.05	14.50	9.2	74.1	44.9	11.1	22.8	2.03
		STD	39.83	1.0	3.38	1.18	1.00	0.15	1.50	2.15	0.23	0.55	0.44
Simeto	2	1	414.5	3	26.67	3.65	12.29	8.9	74.5	43.7	11.8	24.2	1.81
		2	602	4	22.16	4.7	12.17	9.0	77.2	50.8	11.6	23.9	2.07
		3	465.5	5	34.37	3.29	14.33	9.4	69.1	47.8	12.9	26.8	1.98
		Mean	494	4.0	27.73	3.88	12.93	9.1	73.6	47.4	12.1	25.0	1.95
		STD	96.94	1.0	6.17	0.73	1.21	0.26	4.12	3.56	0.70	1.59	0.13
Zühre	3	1	642.5	2	31.57	7.31	16.37	9.3	76.6	55.4	10.4	21.0	2.18
		2	500	3	31.75	3.76	14.26	9.2	71.4	41.3	11.1	22.8	1.81
		3	535.5	1	35.11	7.05	16.57	9.5	77.0	47.0	10.9	22.2	2.09
		Mean	559.3	2.0	32.81	6.04	15.73	9.3	75.0	47.9	10.8	22.0	2.03
		STD	74.18	1.0	1.99	1.98	1.28	0.15	3.12	7.11	0.36	0.92	0.19
Güney yıldızı	4	1	391.5	2	34.39	4.13	17.35	8.9	73.0	41.4	11.7	24.1	1.68
		2	548	3	32.89	6.43	16.88	9.2	74.1	44.8	10.7	21.8	2.06
		3	567.5	4	32.24	5.95	16.24	9.2	78.1	36.1	10.3	20.6	1.85
		Mean	502.3	3	33.17	5.50	16.82	9.1	75.1	40.8	10.9	22.2	1.86
		STD	96.48	1.0	1.10	1.21	0.56	0.17	2.68	4.38	0.72	1.78	0.19
Fırat-93	5	1	458.5	2	31.29	4.04	14.04	8.9	69.8	46.3	12.3	25.6	2.18
		2	527	3	32.53	6.64	15.44	9.2	74.5	41.1	12.9	26.8	2.03
		3	696	3	31.94	6.51	15.37	9.2	80.3	56.0	11.5	23.8	2.15
		Mean	560.5	2.7	31.92	5.73	14.95	9.1	74.9	47.8	12.2	25.4	2.12
		STD	122.24	0.58	0.62	1.47	0.79	0.17	5.26	7.56	0.70	1.51	0.08

Entry name	Entry no	Replica	Frike yield (g/3 kg spikes)	Frike colour (1-5) Visual	L* (Chroma meter)	a* (Chroma meter)	b* (Chroma meter)	Grain moisture (%)	Hectolitre weight (kg/hL)	1000 kernel weight (g)	Protein (%)	Wet gluten (%)	Raw cellulose (%)
R5 (Hom+)	6	1	552.5	1	31.48	6.88	14.40	9.4	77.1	56.6	11.4	23.1	1.41
		2	687.5	3	26.57	5.34	14.65	9.2	73.9	61.7	10.8	22.4	1.75
		Mean	620.03	2	29.03	6.11	14.53	9.3	75.5	59.2	11.1	22.8	1.58
		STD	95.42	1.4	3.47	1.09	0.18	0.14	2.26	3.61	0.42	0.49	0.24
Sariçanak-98	7	1	427	3	34.09	3.23	16.6	8.7	73.5	41.45	12.0	24.8	2.04
		2	396	4	31.00	3.80	14.25	9.3	73.8	37.01	11.3	23.1	0.74
		3	532	5	34.95	5.28	15.24	8.9	80.0	48.46	10.3	20.8	2.01
		Mean	451.7	4.0	33.35	4.10	15.36	9.0	75.8	42.3	11.2	22.9	1.59
		STD	71.28	1.0	2.08	1.06	1.18	0.31	3.67	5.77	0.85	2.01	0.74
Artuklu	8	1	565	1	31.24	5.33	15.09	9.1	77.2	44.5	11.5	23.6	2.34
		2	645	2	32.73	7.16	16.77	9.0	78.4	48.86	11.6	23.9	2.88
		3	570	4	31.09	5.46	14.95	9.4	77.0	47.2	10.4	21.0	2.29
		Mean	593.3	2.3	31.69	5.98	15.60	9.2	77.5	46.9	11.2	22.8	2.50
		STD	44.81	1.53	0.91	1.02	1.01	0.21	0.76	2.20	0.67	1.59	0.33
R5 (Hom-)	9	1	502.8	5	31.85	4.42	14.38	9.4	72.5	50.2	11.3	23.3	1.82
		2	611	4	31.90	6.78	15.44	9.3	74.2	48.0	11.1	22.6	2.14
		Mean	556.9	4.5	31.88	5.60	14.91	9.4	73.4	49.1	11.2	23.0	1.98
		STD	76.50	0.71	0.04	1.67	0.75	0.1	1.2	1.56	0.14	0.49	0.23
R112 (Hom+)	10	1	377.5	4	29.17	5.06	13.08	9.4	75.1	53.04	12.2	25.4	1.65
		2	357	5	30.65	2.93	15.26	9.3	76.3	50.8	11.0	22.5	2.63
		3	282	5	31.93	2.64	12.83	9.0	76.0	47.3	11.7	24.2	1.36
		Mean	338.8	4.7	30.58	3.54	13.72	9.2	75.8	50.4	11.6	24.0	1.88
		STD	50.28	0.58	1.38	1.32	1.34	0.2	0.6	2.9	0.60	1.46	0.67
Sümerli	11	1	620	3	33.14	5.19	16.06	9.1	79.6	55.9	11.5	23.6	1.76
		2	501	3	32.07	3.42	15.63	9.1	73.6	31.9	10.9	22.4	2.03
		3	411	3	32.4	5.29	16.38	9.2	69.6	32.8	10.2	20.5	1.77
		Mean	510.7	3	32.54	4.63	16.02	9.1	74.3	40.2	10.9	22.2	1.85
		STD	104.83	0.00	0.55	1.05	0.38	0.06	5.03	13.60	0.65	1.56	0.15

Entry name	Entry no	Replica	Frike yield (g/3 kg spikes)	Frike colour (1-5) Visual	L* (Chroma meter)	a* (Chroma meter)	b* (Chroma meter)	Grain moisture (%)	Hectolitre weight (kg/hL)	1000 kernel weight (g)	Protein (%)	Wet gluten (%)	Raw cellulose (%)
R112 (Hom-)	12	1	431.5	1	31.73	6.86	16.06	9.0	81.3	54.5	12.1	25.1	1.97
		2	591	1	29.8	6.78	14.61	9.0	77.4	54.7	10.7	21.7	1.99
		3	577	2	32.46	4.64	14.94	9.2	75.8	56.0	12.9	27.0	2.46
		Mean	533.2	1.3	31.33	6.09	15.20	9.1	78.2	55.1	11.9	24.6	2.14
		STD	88.32	0.58	1.37	1.26	0.77	0.12	2.83	0.81	1.11	2.69	0.28
A.Kale-2000	13	1	590	2	33.14	6.34	15.91	9.5	78.4	54.0	11.5	23.6	2.85
		2	416.5	2	35.2	4.69	16.34	9.1	74.4	42.5	10.9	22.4	3.10
		3	658	4	31.82	5.36	16.14	9.1	70.4	38.0	10.7	21.6	2.34
		Mean	554.8	2.7	33.39	5.46	16.13	9.2	74.4	44.8	11.0	22.5	2.76
		STD	124.53	1.15	1.70	0.83	0.22	0.23	4.00	8.25	0.42	1.01	0.39
Edessa	14	1	702	2	30.03	7.33	16.6	9.3	73.0	48.25	11.3	23.1	1.99
		2	622.5	2	27.86	6.46	14.29	9.4	74.2	41.0	11.0	22.4	2.47
		3	539	4	30.46	5.2	14.98	9.2	70.7	41.77	10.9	22.2	2.30
		Mean	621.2	2.7	29.45	6.33	15.29	9.3	72.6	43.7	11.1	22.6	2.25
		STD	81.51	1.15	1.39	1.07	1.19	0.10	1.78	3.98	0.21	0.47	0.25
R23 (Hom-)	15	1	520	3	36.87	5.02	15.56	9.1	74.7	39.6	11.7	24.2	1.96
		2	395.5	3	34.17	3.42	14.98	9.1	62.5	44.2	12.1	25.0	1.97
		3	444	4	29.74	6.63	14.4	9.6	76.2	65.5	10.5	21.4	2.31
		Mean	453.2	3.3	33.59	5.02	14.98	9.3	71.1	49.8	11.4	23.5	2.08
		STD	62.75	0.58	3.60	1.61	0.58	0.29	7.51	13.82	0.83	1.89	0.20
Tüten-2002	16	1	730.3	1	27.99	7.24	15.24	9.1	74.5	49.9	10.5	21.3	2.54
		2	629.5	1	32.19	6.55	16.67	9.2	75.2	52.3	9.8	19.6	1.69
		Mean	679.9	1	30.09	6.90	15.96	9.15	74.8	51.1	10.1	20.5	2.12
		STD	71.28	0.00	2.97	0.49	1.01	0.07	0.48	1.70	0.51	1.20	0.60
Perre	17	1	503	1	34.29	6.61	16.27	9.3	80.9	47.7	10.6	21.0	1.82
		2	557	1	32.00	5.63	15.68	9.5	75.6	36.4	9.7	19.5	1.75
		3	651.5	2	37.45	6.6	16.96	8.9	75.3	57.5	10.9	22.3	2.04
		Mean	570.5	1.3	34.58	6.28	16.30	9.2	77.3	47.2	10.4	20.9	1.87
		STD	75.16	0.58	2.74	0.56	0.64	0.31	3.15	10.56	0.62	1.40	0.15

Entry name	Entry no	Replica	Frike yield (g/3 kg spikes)	Frike colour (1-5) Visual	L* (Chroma meter)	a* (Chroma meter)	b* (Chroma meter)	Grain moisture (%)	Hectolitre weight (kg/hL)	1000 kernel weight (g)	Protein (%)	Wet gluten (%)	Raw cellulose (%)
Burgos	18	1	442	2	34.3	3.79	16.74	9.1	70.0	45.26	11.3	23.1	2.05
		2	404.5	3	30.1	2.93	14.66	8.9	71.8	40.43	11.6	23.9	2.18
		3	522.5	5	30.92	5.35	14.48	9.3	69.5	47.72	10.7	21.8	1.81
		Mean	456.3	3.3	31.77	4.02	15.29	9.1	70.4	44.5	11.2	22.9	2.01
		STD	60.29	1.53	2.23	1.23	1.26	0.20	1.21	3.71	0.46	1.06	0.19
Devedişi	19	1	439	3	39.51	2.6	14.12	9.0	57.3	41.74	10.3	20.8	2.23
		2	390	3	34.98	1.87	14.92	8.9	57.0	45.07	9.4	18.8	2.2
		3	355	4	33.19	1.8	13.29	8.9	56.4	31.55	9.7	19.4	1.87
		Mean	394.7	3.3	35.89	2.09	14.11	8.9	56.9	39.5	9.8	19.7	2.10
		STD	42.19	0.58	3.26	0.44	0.82	0.06	0.46	7.04	0.46	1.03	0.20
Hacı Ali	20	1	513	1	35.46	9.21	15.03	8.9	70.8	34.0	10.5	21.5	2.41
		2	447	3	29.78	5.96	15.35	8.6	71.6	53.2	12.1	24.9	2.28
		3	530	4	31.34	8.3	14.09	8.9	70.8	48.7	11.3	23.1	2.62
		Mean	496.7	2.7	32.19	7.82	14.82	8.8	71.1	45.3	11.3	23.2	2.44
		STD	43.84	1.53	2.93	1.68	0.65	0.17	0.46	10.04	0.80	1.70	0.17
